# Supplementary material for: Lifestyle and Sense of Coherence: A comparative analysis among university students in different areas of knowledge
Source: PLoS One. 2023 Sep 28;18(9):e0288624. doi: 10.1371/journal.pone.0288624 (PMC10538790; doi:10.1371/journal.pone.0288624)
Supplement: Attachment — Submitted filename: Response to Reviewers.docx [file pone.0288624.s001.docx]

Dear Dr. Maria José

A revised version of the manuscript is presented with changes in *red, italic, between Parenthesis ( ), and below each reviewer comment.*

The following points were addressed:

- A rebuttal letter uploaded as a separate file labeled 'Response to Reviewers'.
- A marked-up copy of the manuscript uploaded as a separate file labeled 'Revised Manuscript with Track Changes'.
- An unmarked version of the revised paper without tracked changes, uploaded as a separate file labeled 'Manuscript'.

Journal Requirements:

1. Please ensure that your manuscript meets PLOS ONE's style requirements, including those for file naming. The PLOS ONE style templates can be found at

<https://journals.plos.org/plosone/s/file?id=wjVg/PLOSOne_formatting_sample_main_body.pdf> and 
<https://journals.plos.org/plosone/s/file?id=ba62/PLOSOne_formatting_sample_title_authors_affiliations.pdf>

*(Figure citations are in accordance with file names as shown in the formatting guidelines.)*

2. In your Data Availability statement, you have not specified where the minimal data set underlying the results described in your manuscript can be found. PLOS defines a study's minimal data set as the underlying data used to reach the conclusions drawn in the manuscript and any additional data required to replicate the reported study findings in their entirety. All PLOS journals require that the minimal data set be made fully available. For more information about our data policy, please see <http://journals.plos.org/plosone/s/data-availability>.

Upon re-submitting your revised manuscript, please upload your study’s minimal underlying data set as either Supporting Information files or to a stable, public repository and include the relevant URLs, DOIs, or accession numbers within your revised cover letter. For a list of acceptable repositories, please see <http://journals.plos.org/plosone/s/data-availability#loc-recommended-repositories>. Any potentially identifying patient information must be fully anonymized.

Important: If there are ethical or legal restrictions to sharing your data publicly, please explain these restrictions in detail. Please see our guidelines for more information on what we consider unacceptable restrictions to publicly sharing data: <http://journals.plos.org/plosone/s/data-availability#loc-unacceptable-data-access-restrictions>. Note that it is not acceptable for the authors to be the sole named individuals responsible for ensuring data access.

We will update your Data Availability statement to reflect the information you provide in your cover letter.

*(All data used to support the results are fully available at* [*https://datadryad.org/stash/share/_RlHZJP5BJckyC4EZQ2_Q-Rjtj39aMxSbe-3sLTDhko*](https://datadryad.org/stash/share/_RlHZJP5BJckyC4EZQ2_Q-Rjtj39aMxSbe-3sLTDhko) *and* [*https://doi.org/10.5061/dryad.bcc2fqzhd*](https://doi.org/10.5061/dryad.bcc2fqzhd)*)*

3. We note that you have stated that you will provide repository information for your data at acceptance. Should your manuscript be accepted for publication, we will hold it until you provide the relevant accession numbers or DOIs necessary to access your data. If you wish to make changes to your Data Availability statement, please describe these changes in your cover letter and we will update your Data Availability statement to reflect the information you provide.

*(The changes included in the new version are described in the cover letter.)*

4. Your ethics statement should only appear in the Methods section of your manuscript. If your ethics statement is written in any section besides the Methods, please move it to the Methods section and delete it from any other section. Please ensure that your ethics statement is included in your manuscript, as the ethics statement entered into the online submission form will not be published alongside your manuscript.

*(We moved Ethics statement from “Introduction” to “Material and methods” section.)*

5. Please ensure that you refer to Figure 1 in your text as, if accepted, production will need this reference to link the reader to the figure.

*(We have not obtained permission from the copyright holder, so figure 1 was removed from the manuscript.)*

6. We note that Figure 1 in your submission contain copyrighted images. All PLOS content is published under the Creative Commons Attribution License (CC BY 4.0), which means that the manuscript, images, and Supporting Information files will be freely available online, and any third party is permitted to access, download, copy, distribute, and use these materials in any way, even commercially, with proper attribution. For more information, see our copyright guidelines: <http://journals.plos.org/plosone/s/licenses-and-copyright>.

We require you to either (1) present written permission from the copyright holder to publish these figures specifically under the CC BY 4.0 license, or (2) remove the figures from your submission:

(1) You may seek permission from the original copyright holder of Figure 1 to publish the content specifically under the CC BY 4.0 license.

We recommend that you contact the original copyright holder with the Content Permission Form (<http://journals.plos.org/plosone/s/file?id=7c09/content-permission-form.pdf>) and the following text:
“I request permission for the open-access journal PLOS ONE to publish XXX under the Creative Commons Attribution License (CCAL) CC BY 4.0 (<http://creativecommons.org/licenses/by/4.0/>). Please be aware that this license allows unrestricted use and distribution, even commercially, by third parties. Please reply and provide explicit written permission to publish XXX under a CC BY license and complete the attached form.”

Please upload the completed Content Permission Form or other proof of granted permissions as an ""Other"" file with your submission.

In the figure caption of the copyrighted figure, please include the following text: “Reprinted from [ref] under a CC BY license, with permission from [name of publisher], original copyright [original copyright year].”

(2) If you are unable to obtain permission from the original copyright holder to publish these figures under the CC BY 4.0 license or if the copyright holder’s requirements are incompatible with the CC BY 4.0 license, please either i) remove the figure or ii) supply a replacement figure that complies with the CC BY 4.0 license. Please check copyright information on all replacement figures and update the figure caption with source information. If applicable, please specify in the figure caption text when a figure is similar but not identical to the original image and is therefore for illustrative purposes only.

*(We removed the figure.)*

7. Please review your reference list to ensure that it is complete and correct. If you have cited papers that have been retracted, please include the rationale for doing so in the manuscript text, or remove these references and replace them with relevant current references. Any changes to the reference list should be mentioned in the rebuttal letter that accompanies your revised manuscript. If you need to cite a retracted article, indicate the article’s retracted status in the References list and also include a citation and full reference for the retraction notice.

*(All references were checked. The new version also includes a general reference updating.*

*References*

- *Updated but keeping the same author: 1, 3 and 17.*
- *Checked: 2, 4,6,7, 8,11, 12, 13, 14, 16, 18, 20, 21, 22, 24, 25, 26, 29, 31,32,33, 34, 35, 36, 38, 39, 41, 42, 43, 45, 46 and 47.*
- *Deleted and replaced with more updated ones: 5,11 and 15.*
- *Added: 9,10,15, 19,23, 27, 28, 30, 37, 40, 44, 48 and 49.)*

8. Review Comments to the Author

Please use the space provided to explain your answers to the questions above. You may also include additional comments for the author, including concerns about dual publication, research ethics, or publication ethics. (Please upload your review as an attachment if it exceeds 20,000 characters)

Reviewer #1: Maybe it will be good to have some references regarding cultural validation about this subject.

*(We have included the most important studies conducted in Brazil in different social context of Lifestyle and Sense of Coherence. The following reference numbers are directly associated with these approach: 6,9,10, 12, 13, 14, 16, 24, 29, 32, 33, 34, 41 and 45. These references provide valuable insights into the cultural context to the overall validation of our research.)*

Very interesting but maybe the subject is very limited to the reality of a small circle.
Some references must be actualized.

*(We would like to point out that family medicine and health community is more particularly engaged with domains covered by FANTASTICO questionnaire. Despite the concept of salutogenesis and sense of coherence may not be commonly used in medical intervention, these aspects have been increasingly emphasized as modifiable risk factors to many diseases and how people are committed to heathcare.*

*We hope to add to the body of knowledge by examining these domains and offer new insights to healthcare practice guidelines.)*

Reviewer #2: Congratulations on the manuscript, pertinence, work done, and clarity.
Introduction: well structured and with the appropriate items
Method: makes reference to aspects of scientific and methodological rigor
Mention of ethical aspects
Excellent discussion of the data, however the references should be updated.
Bibliography: Update references - a great percentage are more than 10 years

*(We have now included more recent and significant studies described in the literature with more than 60% of references published in the last six years. These references are in line with more recent understanding about the current state of research in the field. We believe this update considerably improved the quality of our research.)*

**Additional Editor Comments:**

Dear Author, your work has been reviewed and it is considered that minor changes are necessary.

The work is relevant and within the scope of the journal.
It is well-founded and has a robust statistical treatment.
The literature review should be improved with more recent references.


[Note: HTML markup is below. Please do not edit.]

Reviewers' comments:

Reviewer's Responses to Questions

**Comments to the Author**

1. Is the manuscript technically sound, and do the data support the conclusions?

The manuscript must describe a technically sound piece of scientific research with data that supports the conclusions. Experiments must have been conducted rigorously, with appropriate controls, replication, and sample sizes. The conclusions must be drawn appropriately based on the data presented.

Reviewer #1: Yes

Reviewer #2: Yes

2. Has the statistical analysis been performed appropriately and rigorously?

Reviewer #1: Yes

Reviewer #2: Yes

3. Have the authors made all data underlying the findings in their manuscript fully available?

The [PLOS Data policy](http://www.plosone.org/static/policies.action#sharing) requires authors to make all data underlying the findings described in their manuscript fully available without restriction, with rare exception (please refer to the Data Availability Statement in the manuscript PDF file). The data should be provided as part of the manuscript or its supporting information, or deposited to a public repository. For example, in addition to summary statistics, the data points behind means, medians and variance measures should be available. If there are restrictions on publicly sharing data—e.g. participant privacy or use of data from a third party—those must be specified.

Reviewer #1: Yes

Reviewer #2: Yes

4. Is the manuscript presented in an intelligible fashion and written in standard English?

PLOS ONE does not copyedit accepted manuscripts, so the language in submitted articles must be clear, correct, and unambiguous. Any typographical or grammatical errors should be corrected at revision, so please note any specific errors here.

Reviewer #1: Yes

Reviewer #2: Yes

5. Review Comments to the Author

Please use the space provided to explain your answers to the questions above. You may also include additional comments for the author, including concerns about dual publication, research ethics, or publication ethics. (Please upload your review as an attachment if it exceeds 20,000 characters)

Reviewer #1: Maybe it will be good to have some references regarding cultural validation about this subjet.
Very interesting but maybe the subjeto is very limited to the reality of a small circle.
Some references must be actualized.

Reviewer #2: Congratulations on the manuscript, pertinence, work done, and clarity.
Introduction: well structured and with the appropriate items
Method: makes reference to aspects of scientific and methodological rigor
Mention of ethical aspects
Excellent discussion of the data, however the references should be updated.
Bibliography: Update references - a great percentage are more than 10 years

6. PLOS authors have the option to publish the peer review history of their article ([what does this mean?](https://journals.plos.org/plosone/s/editorial-and-peer-review-process#loc-peer-review-history)). If published, this will include your full peer review and any attached files.


If you choose “no”, your identity will remain anonymous but your review may still be made public.


**Do you want your identity to be public for this peer review?** For information about this choice, including consent withdrawal, please see our [Privacy Policy](https://www.plos.org/privacy-policy).

Reviewer #1: **Yes:** Raul Alberto Carrilho Cordeiro

Reviewer #2: No

[NOTE: If reviewer comments were submitted as an attachment file, they will be attached to this email and accessible via the submission site. Please log into your account, locate the manuscript record, and check for the action link "View Attachments". If this link does not appear, there are no attachment files.]

While revising your submission, please upload your figure files to the Preflight Analysis and Conversion Engine (PACE) digital diagnostic tool, <https://pacev2.apexcovantage.com/>. PACE helps ensure that figures meet PLOS requirements. To use PACE, you must first register as a user. Registration is free. Then, login and navigate to the UPLOAD tab, where you will find detailed instructions on how to use the tool. If you encounter any issues or have any questions when using PACE, please email PLOS at [figures@plos.org](mailto:figures@plos.org). Please note that Supporting Information files do not need this step.

*(We uploaded the figure files to PACE digital diagnostic tool, in order to ensure that figures meet PLOS requirements.)*
